# The European Biophysics Journal Prizes 2025: Recognising biophysical science at sub-cellular, cellular and tissue levels of organisation

**DOI:** 10.1007/s00249-026-01822-x

**Published:** 2026-02-06

**Authors:** Robert J. C. Gilbert

**Affiliations:** Division of Structural Biology, Centre for Human Genetics, University of Oxford, Roosevelt Drive, Oxford, OX3 7BN UK

**Keywords:** Small-angle X-ray scattering, Synaptic vesicles, Mechanosensing, Cell membrane ion channels, Ciliary beating, Metachronal waves and transport

## Abstract

The European Biophysics Journal Prizes for 2025, awarded at the European Biophysical Societies Association (EBSA) Congress in Rome last July, recognised three of the outstanding papers published by the Journal in 2022 and 2023. In making these awards, the Journal and EBSA again sought to recognise publications which were already proving their impact. The studies recognised are diverse and highlight the vitality of biophysical science in the 2020s. They concern studies of (i) synaptic vesicles undergoing glutamate uptake, (ii) red blood cells under pressure and the effects of mechanically triggered calcium influx, and (iii) how ciliary beating, the resulting wave effects and downstream transport processes interrelate in trachea. As such these Prizes recognise excellent biophysical science at sub-cellular, cellular and tissue levels of organisation.

## Main text

The three papers recognised by the European Biophysics Journal Prizes for 2025 were by (i) Komorowski et al. and Salditt, a study using small-angle X-ray scattering (SAXS) of synaptic vesicles, with a particular focus on the activity and even location of glutamate uptake transporters (Komorowski et al. 2022); (ii) Wang et al. and Ju, a study of the uptake of calcium by red blood cells under different pressure regimes, leading to downstream cell shrinkage due to potassium and water efflux (Wang et al. 2022a); and (iii) Burn et al. and Frenz, a study using video microscopy to explore the relationship of tracheal ciliary beating frequency (CBF), metachronal (characteristic coordinated ciliary) wavelength, metachronal wave propagation velocity, and mucociliary transport – to find unexpected and dramatic differences in mechanism and speed/efficiency of transport between the trachea of mammals and birds (Burn et al. 2022). These studies showcase biophysics at the sub-cellular (even molecular), cellular and tissue level (Pajic-Lijakovic and Milivojevic 2023; Pajic-Lijakovic et al. 2024), and so neatly highlight the field’s impact at different scales.

### Synaptic vesicles: tackling heterogeneity in a dispersed system

A fundamental challenge for biophysics is how to understand the organisational features of heterogeneous systems, which may be characteristic of them and even necessary to their function (Mehra and Kepp 2022; Tortorella et al. 2022). Characterisation of such systems is necessary if we are to understand their mechanistic role – which may be a molecular mechanism, as with the heterogeneity of some ion channel species (Plakhova 2025), or a cellular mechanism as with vesicles, exosomes, and the like (Parisse et al. 2017). Synaptic vesicles are heterogeneous in size, compositionally and conformationally – including the location of their distinctive resident proteins, such as glutamate transporters (Komorowski et al. 2022).

**Figure. 1:**
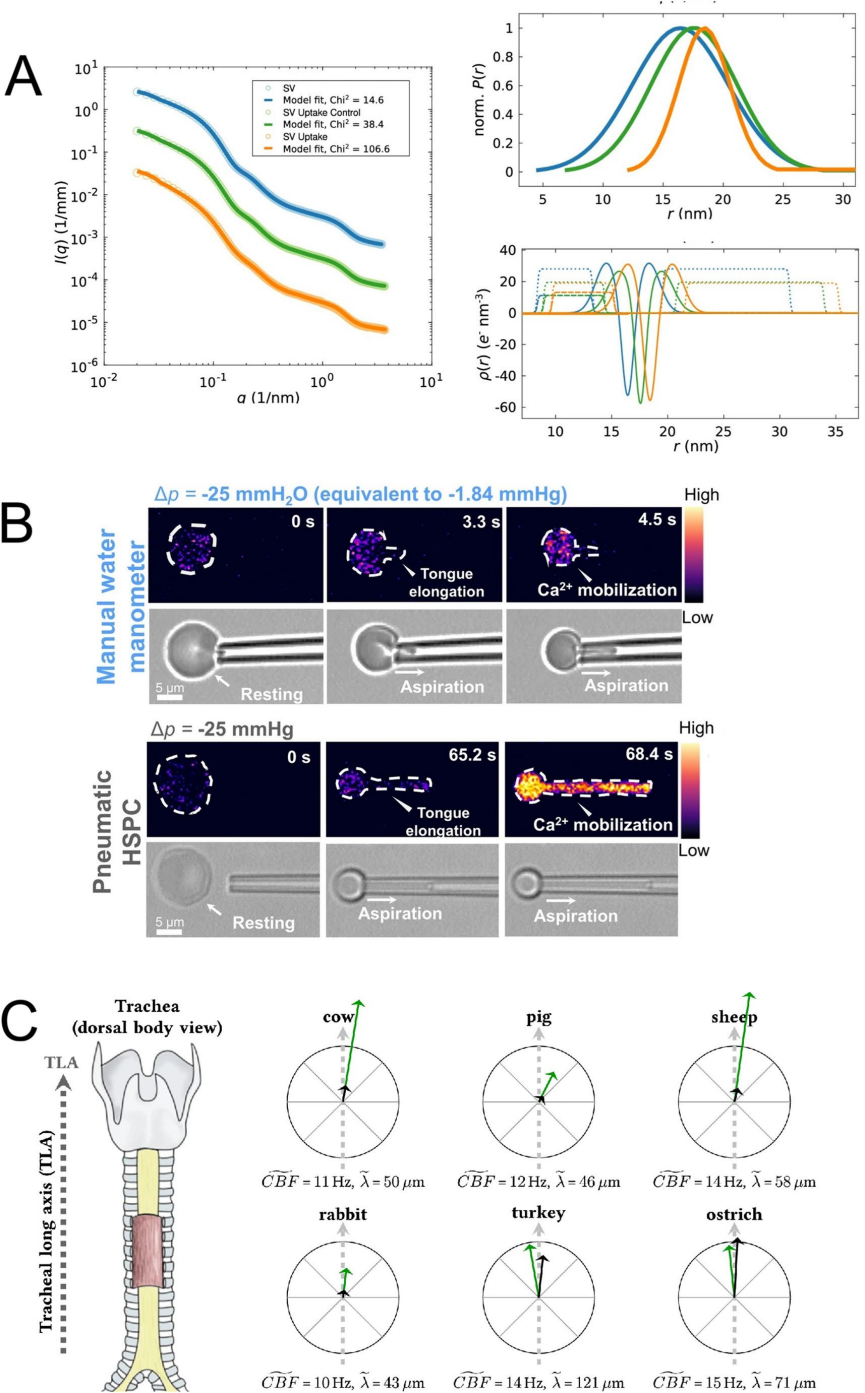
(**A)** Small-angle X-ray scattering (SAXS) studies of structural changes in synaptic vesicles (SVs) arising from neurotransmitter (glutamate) uptake. *Left* SAXS data and anisotropic-SV model fits for SV only (blue), an SV uptake control (green) and SV uptake state (orange). *Top right* normalised Gaussian size distributions from the least-square fits shown *left*. *Bottom right* electron density profiles: of the lipid bilayer (solid line), of local Gaussian chains (modelling resident proteins) (dotted line) and of Gaussian chains spherically averaged (dashed line). See [1] for more information. **(B)**: *Top* snapshots of aspiration of a red blood cell by a manual water manometer, showing a slight increase in Cal-520 intensity after 4.5 seconds of aspiration by -25 mmH_2_O pressure (equivalent -1.84 mmHg). The right hand colour bar indicates the normalised calcium-dependent intensity level. *Bottom* snapshots of pneumatic aspiration of a red blood cell, with a significant Cal-520 increase observed when aspirated by -25mmHg, and with again the right hand colour bar indicating the normalised calcium-dependent intensity level. See [2] for more information. **(C)**: A schematic diagram illustrating the results presented by [3]. All examined tracheal explants were derived from the centre of the anterior tracheal wall (*left*, as marked). The six black and green arrows shown within the crosshairs on the *right* represent the mucociliary transport velocity (black) and the wave propagation velocity (green). For each crosshair, the radius indicates 200 μm/s. Therefore, the magnitudes and the directions can directly be compared between species. Furthermore, the numerical values for the median ciliary beating frequency (CBF) and the median wavelength is provided for each species. All images are provided under a Creative Commons Attribution 4.0 International License (http://creativecommons.org/licenses/by/4.0/).

Komorowski et al.’s achievement was twofold. Firstly, they used SAXS to characterise both the size distribution and the more detailed structure of the synaptic vesicles they were studying, in conditions supporting the active uptake of neurotransmitter glutamate (Komorowski et al. 2022). They found that electron density profile analysis – one of the most powerful applications of both SAXS (Szathmary et al. 2025) and small-angle neutron scattering too (Kaltenegger et al. 2025) – provided evidence of conformational changes in synaptic vesicular membrane proteins and of changes in bilayer structure, which might also include protein positional changes or protein conformational changes (Fig. 1A). But a key strength of the paper is the recognition that SAXS as such can only go so far and that other, complementary and related techniques have much to offer towards a more detailed characterisation. It is consistently noted in biophysics that combinations of techniques – even to answer a simple question such as, Do these two molecular species interact? – are most valuable and really should be necessary to a proper understanding (Demeler et al. 2023; Walter and Bepperling 2025).

One obvious such method in the present context is serial X-ray diffraction with single free electron laser pulses (XFEL). Such sources are relatively hard to access – though new developments are proceeding apace (Santis 2025) – but in preparation for such a future study of synaptic vesicles, Komorowski et al. carried out a simulation to show that it is in principle feasible to obtain direct information even on the relative positions and dimensions of vesicle proteins, using such an approach. XFEL data would be, by definition, based on the individual imaging of synaptic vesicles, and the feasibility explored in this paper should pave the way for this stunning kind of study of a sub-cellular functionally-relevant system state. In any case, Komorowski et al. highlight two different ways to address heterogeneity (Komorowski et al. 2022): look at the regions of a structure which are common, though their larger-scale distribution may be heterogeneous, or look at individual cases (as modern ensemble cryo-EM does too, in its own way).

### Pressure sensing in cell membranes: direct observation of downstream effects

The background to the study published by Wang et al. and Ju (Wang et al. 2022a) is the activity of mechanosensitive calcium channels (such as Piezo1), which are key transducers of mechanical stimuli and downstream regulate red blood cell volume (and the volume of other cells too) through the calcium-activated potassium channel K_Ca3.1_, among others (Wang et al. 2022a). In the case of red blood cells, cell volume and deformability will then be impacted by changes in potassium levels, with efflux producing cell shrinkage which physiologically then contributes to cell clearance (Albuisson et al. 2013; Glogowska et al. 2015). The authors thus needed to identify and apply a method which directly examines calcium mobilisation under controlled mechanical forces, to enable them to characterize and quantify the effect of the aspiration force in a physiologically meaningful framework.

To this end, Wang et al. developed a fluorescence-coupled micropipette aspiration technique, enabling them to observe how mechanical effects impact on calcium entry at a single-cell level (Wang et al. 2022a). To differentiate the effects of pressure they made use of two different aspiration-inducing devices: a water manometer (applying -2.94 to 0 mmH_2_O) and a pneumatic high-speed pressure clamp (-25 to 0 mmH_2_O) with calcium influx detected via the calcium-sensitive dye, Cal-520 (Fig. 1B). In this way the authors were able to calculate the (changing/changed) intracellular calcium concentration under controlled conditions, and what is more to visualise changes in morphology at the same time (Wang et al. 2022a).

Immediately after aspiration – inducing the membrane pressure change – responses were observed in calcium levels, but with the water manometer only to a 1.1 × higher fluorescence than before, while the pressure clamp gave a three-times increase. Trialling their device, Wang et al. were able to identify an upper effective limit for the manometer effect, and to find the best out of seven potential segmentation algorithms to allow the signal from the red blood cell only to be acquired.

This study helps to provide a number of insights useful for further work on this and related systems. Firstly, that the water manometer would be a useful tool for cellular manipulation with minimal pre-activation of pressure-induced effects; and then in turn that the pressure clamp is useful as a tool for the direct application of pressures to investigate their impact on intracellular changes relating to pressure effects and to probe mechanosensing in real time (Wang et al. 2022a). Strikingly, Wang et al. found that increased aspiration (mechanical strain) can trigger activation of calcium mechanosensitive channels to increase the internal concentration of calcium by 100 × the normal physiological level. The approach taken by the authors further has the obvious capacity to work complementarily with biomembrane force probe (Wang et al. 2022b) or optical tweezer studies (Faudry et al. 2025; Konyshev et al. 2025).

### Ciliary waves and transport effects: differing architectures and pathways

In their paper Burn et al. and Frenz (Burn et al. 2022) were performing biophysical studies at a tissue level, using high-speed video reflection microscopy to image and characterise in quantitative terms the metachronal wave field (the field arising from ciliary motion) and the resulting mucociliary response: movement. The aim of their work was (i) to establish a general model applicable in all species for the relationship between the mucociliary wave field and mucociliary transport; and (ii) conducted in the belief that mucociliary transport would consistently follow the tracheal longitudinal axis. In fact what they found was that the cross-species model they sought does not exist because of fundamental differences in wave generation, wave alignment with the trachea, and the relationship of the metachronal wave to its movement of material. At the most basic level, they found that the relationship between ciliary movement and the ultimate mucociliary transport is fundamentally different in birds from in mammals (Burn et al. 2022).

The study had to overcome a basic difficulty, which was that Burn et al. needed to record the ciliary movement in ex vivo samples which still make observation of ciliary movement and its effects difficult, and that they were seeking to address a question which had otherwise been considered from the study of samples in a medium under a coverslip (e.g. (Gheber and Priel 1994)), but if the coordination of ciliary motion is, as has been proposed, the result of hydrodynamic coupling through the viscoelastic working medium (Gheber and Priel 1994; Brumley et al. 2014; Elgeti and Gompper 2013) then the medium used fundamentally matters to the activity observed. So Burn et al. needed to study mucociliary transport and the space–time structure of the metachronal wave field in tissues as unaltered as possible by excision and in as close-to-native conditions as possible.

Four mammalian and two bird species were studied, and for obvious reasons it was important to ensure that the gross anatomical orientation of the samples was carefully handled to ensure an accurate understanding of the relationship between ciliary movement, the metachronal wave and the resulting movement of material (Fig. 1C). By working at an air–liquid interface, Burn et al. were able to observe modulation of the mucus surface with reflection microscopy, and measured (i) ciliary beating frequency, (ii) metachronal wavelength, (iii) metachronal wave propagation velocity and (iv) the resulting mucociliary transport velocity (Burn et al. 2022).

What the authors fundamentally do find is that in all mammals (studied) the metachronal ciliary transport deviates from the tracheal long axis, instead following a left-handed helical trajectory; whereas in avian species the two are essentially aligned. Furthermore, avian species show extraordinarily high mucociliary transport speeds, three-to-five times those found in mammals (Fig. 1C). In addition, the bird/mammal difference must be considered even more dramatic when looking at clearance efficacy (Burn et al. 2022). This may presumably be due to the more straightforward trajectory of the avian metachronal ciliary transport compared to the helical path of the mammalian form.

Nevertheless, the actual process of mucociliary transport direction essentially aligns with the direction of the metachronal wave in each case, with a slight tendency to negative values (moving in the wake of the wave). Still, clearly wave movement and object transport correlate, which is as it should be. But, strikingly, the metachronal waves propagate at 4–8 × the speed of the transport in mammals whereas in avian species the wave propagation speed is about the same as the transport speed – which again (may) make sense because of the helical trajectory of the wave around the long axis of the trachea in mammals (Burn et al. 2022).

Why might birds and mammals show such differing relationships between ciliary beating, metachronal waves and the transport of substances by them? It is pointed out that birds ventilate their lungs using air sacs and that their tracheas are on average 2.7 × longer and 1.3 × wider than comparably-sized mammals (Burn et al. 2022). But as noted at the outset of the study, important (other) factors to bear in mind are the way in which metachronal waves are thought to self-organise out of local hydrodynamics, and the impact of distinctive mucosal properties. In addition to such rheological conditions, geometric factors such as ciliary spacing, ciliary orientation, proportion and distribution of ciliate cells and depth of the mucus and the periciliary layer of lower viscosity fluid that lies beneath it are all relevant factors (Burn et al. 2022).

## Conclusion

As noted above, these three fine studies showcase biophysics at the subcellular, cellular and tissue levels and thus the power of the science celebrated and published by the European Biophysics Journal to illuminate the bases of fundamental biological processes.

## Data Availability

No datasets were generated or analysed during the current study.
